# Generation of Aptamers with an Expanded Chemical Repertoire

**DOI:** 10.3390/molecules200916643

**Published:** 2015-09-14

**Authors:** Stella Diafa, Marcel Hollenstein

**Affiliations:** Department of Chemistry and Biochemistry, University of Bern, Freiestrasse 3, CH-3012 Bern, Switzerland; E-Mail: stella.diafa@dcb.unibe.ch

**Keywords:** aptamers, modified nucleoside triphosphates, polymerases, SELEX, therapeutic oligonucleotides, chemically modified nucleic acids, synthetic genetic polymers

## Abstract

The enzymatic co-polymerization of modified nucleoside triphosphates (dN*TPs and N*TPs) is a versatile method for the expansion and exploration of expanded chemical space in SELEX and related combinatorial methods of *in vitro* selection. This strategy can be exploited to generate aptamers with improved or hitherto unknown properties. In this review, we discuss the nature of the functionalities appended to nucleoside triphosphates and their impact on selection experiments. The properties of the resulting modified aptamers will be described, particularly those integrated in the fields of biomolecular diagnostics, therapeutics, and in the expansion of genetic systems (XNAs).

## 1. Introduction

**Figure 1:**
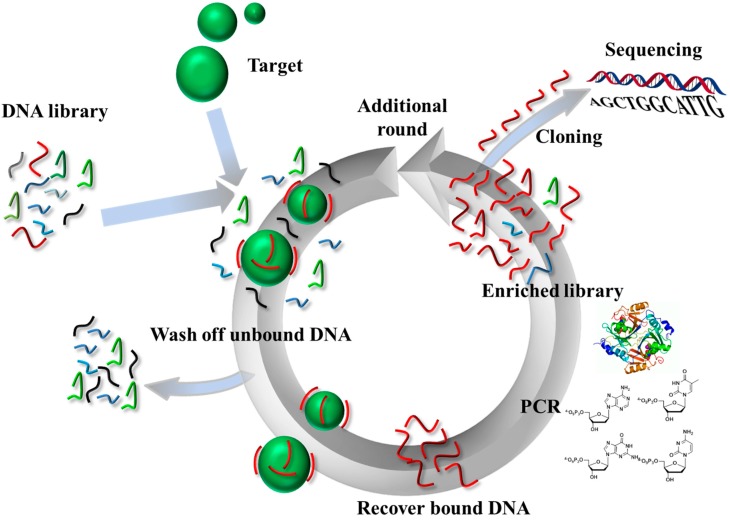
Schematic representation of the SELEX strategy for the identification of DNA aptamers.

Aptamers are single-stranded DNA or RNA molecules that bind to specific targets with high affinity and are often considered to be the nucleic acids’ pendant of antibodies [1,2]. The advent of aptamers was propelled by the discovery of SELEX [3,4,5] (Systematic Evolution of Ligands by Exponential enrichment) and related combinatorial methods of *in vitro* selection. In SELEX, large populations of oligonucleotides (typically ~10^14^ molecules of up to 100 nucleotides in length) are screened for their potential binding affinity for a defined target (see Figure 1) [6,7]. In this chemical variant of Darwinian evolution, the initial population of oligonucleotides is bound to the selected target and only the species capable of binding are retained, PCR-amplified, and used for subsequent rounds of selection [8]. By modulating various parameters of the selection experiment, including the nature of the target, the length of the randomized region of the original library, and the selection stringency, a broad array of multifunctional aptamers can be obtained. Moreover, since the inception of the traditional SELEX method in 1990, numerous modifications and variants have been developed to fit the choice of the target, the different conditions, and include new technologies such as high-throughput sequencing methods and microfluidics [9,10].

While natural (unmodified) nucleic acids are capable of folding into intricate three-dimensional structures and bind to their target by shape complementarity, the lack of chemical modifications present in these functional nucleic acids was soon recognized to be detrimental for their potential *in vivo* applications. Indeed, as is the case for any wild-type DNAs and RNAs, aptamers are prone to nuclease degradation and are sensitive to the chemical (e.g., basic media, divalent metal cations) or physical (e.g., heat) environment [11,12,13]. Furthermore, nucleic acids are rather functionality deprived biopolymers, especially when compared to their proteinaceous counterparts [14]. This dearth of functional groups limits aptamers in their capacity to form specific interactions with more demanding targets and might preclude the formation of hydrogen bonding patterns or hydrophobic pockets as seen in antibodies and proteins in general. These limitations were recognized shortly after the advent of SELEX and modified aptamers can be obtained either (1) by introducing modifications into the scaffold of selected aptamers via standard solid-phase synthesis or (2) by using modified nucleoside triphosphates (dN*TPs and N*TPs) directly in the selection process [2,15,16,17].

In this context, this review will focus specifically on the burgeoning field of modified aptamers. A strong emphasis will be given on aptamers obtained by *in vitro* selection with modified nucleoside triphosphates (dN*TPs and N*TPs) to increase their binding efficiencies, selectivities, and their pharmacokinetic properties. Moreover, their application for therapeutic and diagnostic purposes will be discussed as well as aptamers made of synthetic genetic polymers (XNAs) [18,19,20]. Many excellent review articles [2,21] have addressed the post-synthetic modification and labelling of aptamers and this vast topic is beyond the scope of the present review.

## 2. Modified Aptamers for Therapeutic and Diagnostic Applications

A large number of distinctive properties renders aptamers attractive candidates for therapeutic applications [22,23]. For example, their high affinity and specificity for their targets is comparable to that of proteinaceous antibodies, however, unlike the latter, their size is smaller (8–15 kDa) and they lack immunogenicity. Furthermore, access to aptamers has been facilitated by recent progress in standard automated solid-phase synthesis and larger quantities can be synthesized at low cost (less than 200 $/g for unmodified aptamer; up to 1 kg scale) [24,25]. Modulation of the nature of the target in the selection protocol allows for the generation of aptamers against a great variety of targets ranging from small molecules and peptides to proteins and cells. Interestingly, their inhibitory action can be reversed by an antidote, a feature that holds great potential for drug design [26,27]. Indeed, the shape and three-dimensional structure of an aptamer can be distorted by binding to a polymer or complementary oligonucleotides, which in turn alters the activity of the aptamer [28,29,30]. This approach has recently been used to modulate the spatiotemporal activity of an anti-nucleolin aptamer both *in vivo* and *in vitro* by using a complementary oligonucleotide antidote equipped with photocleavable linkages [30]. In a slightly different approach, aptamers can be selected against known drugs and their binding affinity then serves as the basis for the antidote effect. In this context, an aptamer was selected against bivalirudin, an anticoagulant drug, and shown to cause a dose-dependent regeneration of the clotting activity in the presence of bivalirudin [31].

Taken together, aptamers are very potent and versatile ligands and display a multitude of favorable assets for their use as diagnostic tools and therapeutic agents. Currently, one anti-VEGF aptamer has been approved for the treatment of age-related macular degeneration [32], whilst numerous aptamers are in pre-clinical studies and clinical trials for a broad variety of applications including gene therapy [33,34,35], immunotherapy [36,37], cancer therapy [27,38], and the development of imaging agents [39,40].

However, despite their numerous favorable characteristics, aptamers suffer from several drawbacks: (i) while aptamers are stable under long-term storage conditions, their biostability is compromised by rapid renal filtration and by the presence of nucleases; (ii) nucleic acids have a rather depleted chemical arsenal and thus lack functional groups that could enhance the binding affinity to more difficult targets (e.g., single enantiomers of small organic molecules or glycosylated proteins) and favor the formation of additional potential interactions with the target; (iii) with the exception of the 3′- and 5′-termini, the chemical modification of aptamers via solid-phase synthesis can lead to a depletion of the binding affinity; (iv) some selection experiments yield aptamers with poor binding affinities as a result of either lack of functionalities to sustain strong binding or a competition between amplification in SELEX and functional fitness of the sequences. In order to (partially) alleviate these shortcomings, modifications located at the level of the sugar unit, the nucleobase, or the backbone of the constituting nucleotides can be introduced using dN*TPs as vectors in selection experiments. However, for dN*TPs to be acceptable candidates in SELEX, they obligatorily must be good substrates for polymerases, and the resulting modified sequences need to serve as templates for the conversion into wild-type DNA under PCR conditions [11,41]. Recent advances in protein engineering and the development of the compartmentalized self-replication (CSR) and compartmentalized self-tagging (CST) strategies [42,43], have allowed for the evolution of numerous polymerases with expanded substrate tolerance [44,45]. Therefore, finding conditions for the successful polymerization of particular (d)N*TPs can readily be achieved by assessing the substrate acceptance using a pool of engineered and/or evolved polymerases.

The importance and the nature of the functionalities appended on the nucleoside triphosphates along with their use in SELEX will be highlighted through some recent implementations presented in the pages to follow.

### 2.1. Sugar Modifications

**Figure 2:**
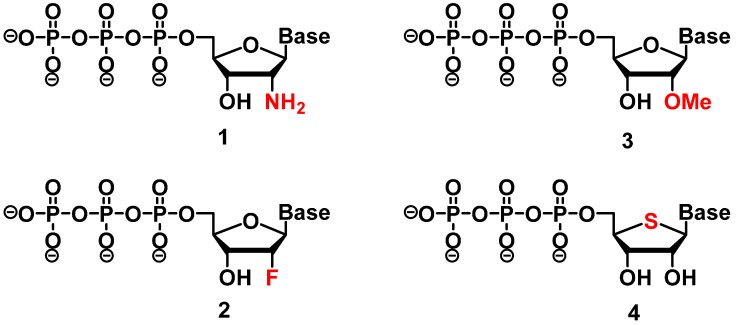
Chemical structures of 2′-modified nucleotides used in selection experiments to generate aptamers with enhanced pharmacokinetic properties: 2′-amino-NTPs **1**, 2′-fluoro-NTPs **2**, 2′-methoxy-NTPs **3**, and 4′-thio-NTPs **4**.

Early modifications of the scaffold of aptamers paralleled progresses attained in the field of antisense research and focused mostly on the 2′-position of the (deoxy-)ribosesugar unit (Figure 2) [1,46]. Initial efforts included 2′-amino pyrimidines **1** [15,47,48,49,50,51,52], 2′-fluoro pyrimidines **2** [16,50,53,54,55] and 2′-methoxy nucleotides **3** [56,57]. All these selection experiments led to the isolation of 2′-modified aptamers that displayed good binding affinities (*K*_d_ values in the low nM down to the pM range) and strong nuclease resistance. In most cases, the isolated aptamers were converted post-selection into shorter and more potent species by solid-phase synthesis.

The first use of N*TPs in a Darwinian *in vitro* selection experiment was reported by Jayasena *et al.* in 1994. The use of 2′-NH_2_-pyrimidine NTPs in the selection experiment instead of their natural counterparts resulted in the isolation of anti-human neutrophil elastase aptamers with good binding affinities (*K*_d_ values in the low nM range) and nuclease resistance (*t*_1/2_ = 20 h in human serum compared to ≤8 min for the unmodified oligonucleotide) [47]. However, despite initial success (e.g., an anti-bFGF (basic fibroblast growth factor) aptamer that displayed a high specificity and a very strong binding affinity (*K*_d_ = 35 pM) for its target [15]), 2′-NH_2_-pyrimidines are only rarely used nowadays due to problems encountered during solid-phase synthesis and their preference for the C2′-endo ribose conformation [11,58]. Nevertheless, in a very elegant approach, Bugaut *et al.* used the 2′-amino-modification as a synthetic handle for the introduction of transient functionalities via imine formation, thus combining aspects of SELEX and dynamic combinatorial chemistry (DCC). This approach was succesfully applied to the selection of anti-TAR modified RNA aptamers [59].

On the other hand, the other two modifications (2′-methoxy and 2′-fluoro) have become the most widely used N*TPs for the *in vitro* evolution of aptamers. The combined use of 2′-fluoro-pyrimidines N*TPs and wild-type purine NTPs in a selection experiment, followed by the post-selection modification and conversion of the unmodified ribonucleotides to 2′-methoxy modifications, led to the isolation, development, and ultimately the approval of pegaptanib (Macugen^®^, Pfizer, New York, NY, USA), the only aptamer-based drug currently approved by the FDA [32,54]. Pegaptanib is a 27 nucleotide long RNA aptamer that is administered for the treatment of neovascular age-related macular degeneration (AMD). It binds to the abundant isoform of the human vascular endothelial growth factor (VEGF) with very high affinity (*K*_d_ = 49 pM), and thus inhibits the interaction with its receptors [24,27].

In a more recent study, Li *et al.* selected RNA aptamers targeting the four members of the epidermal growth factor receptor (EGFR) family starting from an initial RNA pool obtained by the T7 RNA polymerase-mediated transcription with natural and 2′-fluoropyrimidine-modified NTPs [60]. The initial selection experiment led to the isolation of one particular aptamer that displayed a strong binding affinity to human EGFR (*K*_d_ ~40 nM) and was much shorter than the initial randomized region (51 *vs.* 62 nucleotides, respectively). This sequence was then used as a template for a second *in vitro* selection experiment which led to the isolation of aptamer E07. The E07 aptamer had a strong binding affinity for the wild-type receptor comparable to that of the natural substrate EGF (*K*_d_ = 2.4 nM) and was shown to block *in vitro* the EGF-stimulated phosphorylation of the receptor and thus, inhibited the cell proliferation while being also efficiently internalized into EGFR-expressing cells. Finally, aptamer E07 also showed a rather high propensity (*K*_d_ = 36 nM) to bind to the mice form of EGFR (mEGFR) which has 88% homology with the human form [60]. Similarly, a whole cell-SELEX experiment on human non-small-cell lung cancer cells (NSCLC) with 2′-fluoropyrimidine-modified NTPs allowed Esposito *et al.* to isolate a rather short (39 nucleotides) aptamer that could inhibit the epidermal growth factor receptor (EGFR) through tightly binding to the lung cancer cell line A549 (*K*_d_ = 38 nM) and could discriminate these targets from H460 cells [61]. This aptamer was further shown to induce selective cell death both *in vitro* and *in vivo* [61]. Both selection experiments underscore the usefulness of using modified nucleoside triphosphates to generate nuclease-resistant aptamers (the stability of 2′-fluoro-siRNAs has been estimated to be >1 day [62]) that effectively compete with the natural ligand and could induce apoptosis.

The same 2′-fluoro-modification was used by Svobodova *et al.* to generate RNA aptamers against the prostate-specific antigen (PSA), a glycoprotein involved in prostate cancer [63]. One particular aptamer (S2) showed a moderate affinity to the intended target since it bound to PSA with a *K*_d_ value of 630 nM and displayed an 11 nM limit of detection in an apta-PCR assay [63]. The isolated aptamers were also tested for their potential application in both diagnostics and therapeutics, providing an alternative to the well-studied A10 aptamer, another 2′-F-RNA aptamer binding to the prostate-specific membrane antigen (PSMA) [64].

An interesting approach employed *in vivo* SELEX [65] to identify 2′-fluoropyrimidine modified RNA aptamers that were capable of penetrating the blood-brain barrier (BBB) [66]. A 2′-fluoro-modified RNA pool was injected into mice (via tail injection) and the RNA present in the brain was isolated and amplified. After 22 rounds of selection, sequence convergence was observed and one particular sequence, aptamer A15, dominated the RNA population [66]. This aptamer was then shown to specifically accumulate in the brain, rather than in the kidney or liver. Moreover, aptamer A15 was further modified with 2′-methoxy residues to increase the nuclease resistance and tested for brain penetration via *in vitro* internalization assays. It was shown that the aptamer first targets the endothelial cells before entering the brain parenchyma of the A15-injected mice. The biodistribution of the aptamer demonstrated positive signals in numerous brain regions including the cortex, hippocampus, cerebellum, and striatum suggesting successful permeation.

All in all, this versatile modification has been widely exploited over the last years by many groups for various targets (Table 1) and has underlined its significance among other common modifications.

**Table 1:** Summary of the recently generated aptamers using the 2′-fluoro modification.

Aptamer Name	Aptamer Target	*K*_d_ Value (nM)	Reference
E07	Epidermal growth factor receptor (EGFR)	2.4	[60]
CL4	Epidermal growth factor receptor (EGFR)	10	[61]
S2	Prostate-specific antigen (PSA)	630	[63]
A15	Brain penetrating aptamer	-	[66]
R-F t2	NS5B replicase, essential for the replication of hepatitis C virus (HCV)	2.6	[67]
Gint4.T	Platelet-derived growth factor receptor β (PDGFRβ)	9.6	[68]
GL21.T	Transmembrane tyrosine kinase receptor (RTK) Axl	12	[69]
G-3	C-C chemokine receptor type 5 (CCR5)	110	[70]
C26-50	*N*-methyl-d-aspartate (NMDA) receptor ion channel	120	[71]
Apt1	CD44, a cell-surface glycoprotein that serves as a cancer stem cell marker	81.3	[72]
B-68	HIV-1_Ba-L_ glycoprotein 120	52	[73]
GL44	Human U87MG glioma cells	38	[74]
RNA 14-16	p68 RNA helicase, which is involved in colorectal cancer	13,8	[65]
FAIR-6	Interleukin-6 receptor (IL-6R)	40.9	[75]
CD28Apt2, CD28Apt7	CD28 costimulatory receptor for the activation of T lymphocytes	40, 60	[76]
9C7	OX40 costimulatory receptor	1.7	[77]
αV-1, β3-1	αV and β3 subunits of integrin αVβ3	2.7, 6.5	[78]

Unlike 2′-fluoro- and 2′-amino-modified N*TPs, 2′-methoxy-N*TPs have rarely been engaged in selection experiments, despite the very favorable properties of polymers equipped with this particular functional group [56,57]. Most aptamers containing 2′-OMe-units, including pegaptanib (*vide supra*), stem from post-selection engineered sequences obtained from *in vitro* selections that combine 2′-fluoro-pyrimidine N*TPs and wild-type 2′-OH-purine NTPs [56]. The main reason for this relative scarcity resides in the rather poor substrate acceptance of the bulkier 2′-OMe-N*TPs by the wild-type T7 RNA polymerase, especially when compared to the smaller 2′-fluoro- and 2′-amino-modifications [46]. On the other hand, the combination of engineering of mutants of the T7 RNA polymerase [79,80,81,82] and the discovery of optimized conditions [56], have enabled the enzymatic synthesis of 2′-OMe-RNA libraries for their use in *in vitro* selection experiments. In this context, a direct *in vitro* selection experiment using the four modified 2′-OMe-N*TPs along with a small fraction of natural GTP culminated in the isolation of a potent anti-VEGF aptamer (*K*_d_ = 1 nM) [56]. More recently, a combination of three 2′-OMe-N*TPs and the lone, natural dCTP were used in a selection experiment for the generation of an aptamer that binds to the polypeptide tissue factor pathway inhibitor (TFPI) involved in the regulation of the extrinsic coagulation pathway [83]. The resulting aptamer bound selectively and tightly (*K*_d_ = 2.8 nM) to TFPI and was shown to correct thrombin generation in the bleeding disorders hemophilia A and B.

In a very interesting approach, coined cell-uptake selection, three 2′-OMe-N*TPs and the lone, natural GTP were used for the identification of aptamers that could both recognize different cell lines of prostate cancer cells and internalize directly into cells [84]. Indeed, Farokhzad *et al.* rationalized that selecting for aptamers that displayed the highest affinity and selectivity for their targets, for instance by lowering the temperature [85], would not necessarily yield aptameric species capable of being internalized by cells, which is an important prerequisite for potential *in vivo* applications. Consequently, the modified RNA library was first incubated with counter-selection cell lines and the bound species were discarded. The unbound sequences that remained in the supernatant of the counter-selection step were then incubated with the desired cell lines (PC3 and LNCaP cells) at 37 °C. A cell lysis then allowed for the extraction of the RNAs that were capable of internalization [84]. In addition, the stringency of the selection was gradually increased by diminishing both the incubation time and the number of the desired cell lines and by introducing a randomization of the various populations by mutagenic PCR. After 12 rounds of selection, aptamers that recognized and internalized specifically into both cell lines could be identified. Finally, a targeted nanoparticle (NP) encapsulating a chemotherapeutic agent against prostate cancer (docetaxel) was connected to one of the selected 2′-OMe-modified aptamers via maleimide-thiol chemistry and the resulting hybrid construct was then shown to significantly improve the cytotoxicity in the target cells by a combination of aptamer-mediated internalization and a release of the drug [84].

Other sugar modifications have also been utilized in selection experiments for the isolation of aptamers. For instance, in the early 2000s, the group of Matsuda introduced a modification by replacing the 4′-oxygen atom of the sugar unit with a sulfur atom (structure **4** in Figure 2) [86]. The modification was first used for the synthesis of the 4′-thiouridine (4′-thio-UTP) and 4′-thiocytidine (4′-thio-CTP) triphosphates which were then used in the *in vitro* selection of anti-thrombin thioRNA aptamers. The resulting 4′-thio-modified aptamer bound to the thrombin target with high affinity (*K*_d_ = 4.7 nM) and displayed a 50-fold increase in resistance to RNase A as compared to wild-type RNA [86]. This initial selection experiment with two modified 4′-thio-N*TPs was then followed by an optimization of the *in vitro* transcription conditions using the set of four modified NTPs which in turn allowed for the selection of a fully-modified RNA aptamer against the same target [87]. The most potent aptamer resulting from this selection experiment displayed a similar binding affinity to thrombin as the partially modified aptamer (*K*_d_ = 7.2 nM) but presented no sequence homology with the latter [87].

The 4′-thio-modification has also been explored on the DNA level and shown to be compatible with enzymatic synthesis [88,89]. Similarly, selenium modified dNTPs and NTPs have also been synthesized and evaluated for their substrate acceptance by DNA polymerases and T7 RNA polymerase, respectively. In particular, 2′-methylseleno-NTPs [90,91] and 4′-selenothymidine triphosphate (SeTTP) [92] were shown to be readily accepted by their respective polymerases but have not been engaged in any selection experiments so far.

Finally, other sugar-modified nucleoside triphosphates such as LNA, HNA, TNA, and other XNAs have been used as building-blocks for the generation of modified aptamers (*vide infra*) [20].

### 2.2. Backbone Modifications

The backbone of a nucleoside triphosphate can be modified at any of the three phosphorous atoms or the bridging oxygens, but in order to serve as a vector for the introduction of chemical functionalities into nucleic acids, the modifications must be located at the α-phosphorous atom. In this context, phosphorothioate linkages are well established modifications in antisense technology due to their chirality, their stability against nucleases, and their ability to internalize into cells without the help of any carrier [93]. In the context of aptamer selections, the group of Gorenstein has used a two-step selection strategy to obtain thioaptamers as drug carriers that specifically bind to E-Selectin, whose overexpression has been associated with many inflammatory diseases [94]. The selection protocol involved a single α-thio-dATP nucleotide and resulted in the isolation of aptamer ESTA-1 that bound to its target with high affinity (*K*_d_ = 47 nM) without recognizing the other members of the selectin protein family (a *K*_d_ of 13 μM was determined for P-selectin, while no binding to l-selectin could be observed). In addition, the nuclease resistant ESTA-1 inhibited the adhesion of leukocytes (HL-60 cell line) through its binding to E-selectin, which recognizes the carbohydrate ligand sialyl Lewis X expressed by the HL-60 cells [94]. The same group has also previously reported on the selection of mono-thio aptamers against the nuclear factor kappa B (NF-κB; *K*_d_ = 0.8 nM) [95], aptamers acting as inhibitors of the RNase H domain of HIV-1 reverse transcriptase (*K*_d_ = 70 nM for binding to HIV-1 RT) [96], and against CD 44 antibodies (*K*_d_ in the 180–295 nM range) [97], while a fully thio-modified aptamer targeted against an envelope protein of dengue-2 virus was obtained [98].

Recently, a new method for the incorporation of small drug-like molecules at the C5-position of the nucleobase of uridines on a complete monothiophosphate backbone-substituted aptamer in order to obtain next-generation (X-aptamers) with greatly enhanced nuclease resistance and binding affinities has been reported [99]. The group used this method to introduce an ADDA (*N*-acetyl-2,3-dehydro-2-deoxyneuraminic acid) molecule that binds to the hyaluronic acid binding domain of CD44 antibodies (CD44-HABD) and the selected aptamer displayed a binding affinity of less than 10 nM. This work paves the way for the isolation of aptamers with expanded chemical functionalities and combined characteristics of drug molecules, proteins, and nucleic acids.

Even though other backbone modified triphosphates have been reported, including α-*P*-seleno-TTP [100], α-*P*-methyl-TTP [101,102], and α-*P*-borano-α-*P*-thio-TTP [103], none have been employed in the context of SELEX, probably due to their low acceptance by polymerases. However, a notable exception is the α-*P*-borano-modification. Indeed, single α-*P*-borano-modified triphosphates (α-B-UTP and α-B-GTP) were used in the selection of boronated aptamers against ATP [104]. Surprisingly, the selection with α-B-UTP resulted in a population of oligonucleotides that bore very little sequence homology to that obtained with α-B-GTP.

### 2.3. Base Modifications

#### 2.3.1. General Base-Modifications

**Figure 3:**
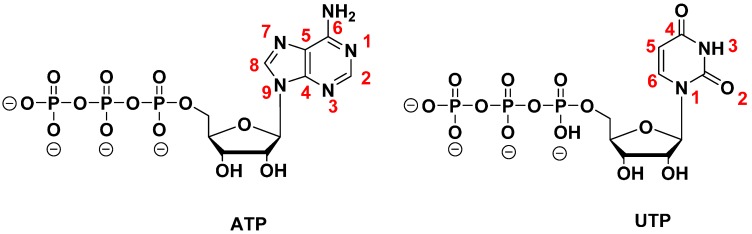
Chemical structure and numbering of the purine and pyrimidine nucleobases exemplified for ATP and UTP.

**Figure 4:**
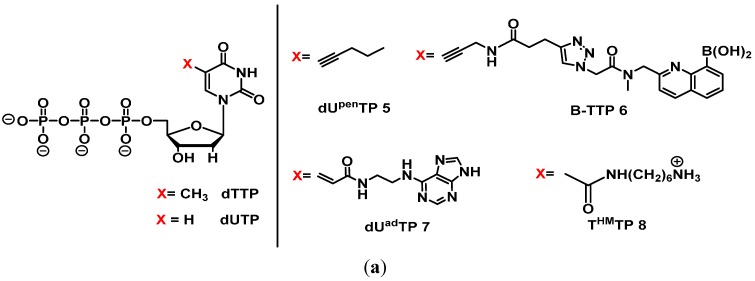

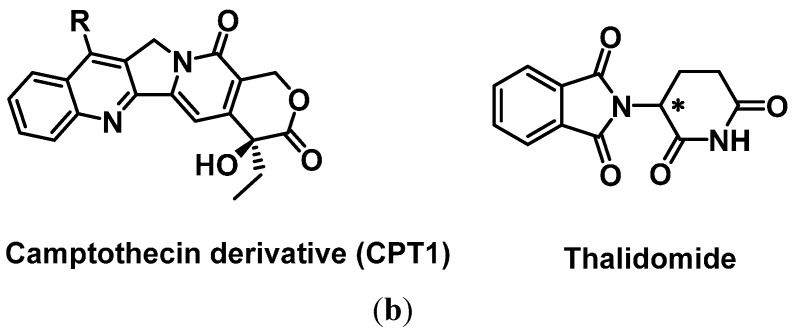
(**a**) Chemical structures of some base-modified dN*TPs used in aptamer selection experiments; (**b**) Target molecules camptothecin and thalidomide.

Modifications of the nucleobases are mostly located at the C5-position of the pyrimidines and the *N*7 of 7-deaza-purines (see Figure 3), as these positions have been shown to be good substrates for polymerases and well tolerated in the major groove of duplexes [105,106,107,108]. The introduction of functionalities at the level of the nucleobase might increase the contact interactions of oligonucleotides and their intended targets and might create additional secondary structures that are not accessible to wild-type nucleic acids and thus enhance the binding affinity. This concept was exploited in an early selection experiment where Latham *et al.* isolated an anti-thrombin aptamer (albeit with a rather poor binding affinity) using a dUTP equipped with a hydrophobic (pentynyl) residue (dU^pen^TP **5**, Figure 4a) [109]. After this initial example, other selection experiments using base-modified (d)NTPs were reported, including the use of the photoactivatable 5-iodo-UTP to generate an anti-HIV-1 Rev protein aptamer [110] and the inclusion of positively charged residues on dUTP [111] and UTP [112] for the isolation of aptamers selective for ATP.

More recently, Li *et al.* introduced a boronic acid moiety (B-TTP **6**, Figure 4a) into thymidine-5′-triphosphate and selected for aptamers that could bind to fibrinogen through specific recognition of the glycosylation site [113]. The isolated aptamers all displayed dissociation constants that lay in the low nM range, while the unmodified DNA pool of the last selection round displayed a ~1000 fold lower affinity (average *K*_d_ = 5 μM). Binding of the aptamers occurred via interactions of the boronic acid moieties and the glycan structures present in fibrinogen, clearly underscoring the usefulness of this modification [113].

Imaizumi *et al.* utilized a DNA library generated by primer extension reaction including a dUTP equipped with an adenine residue tethered to the C5 site of the nucleobase (dU^ad^TP **7**, Figure 4a) [114]. The introduction of this additional nucleobase was thought to convey additional hydrogen bonding patterns and stacking interactions to the modified DNA population. The resulting modified pool was used to isolate aptamers that would bind to the antitumor agent camptothecin derivative 1 (CPT1, Figure 4b). After 11 rounds of selection, a very potent 70-mer aptamer (CMA-70) could be isolated, which was further converted to a shorter 59-mer version (CMA-59) by sequence refinement. Both CMA-70 and CMA-59 displayed strong binding efficiencies to CPT1 with dissociation constants of 39 and 86 nM, respectively. What is more, this aptamer also demonstrated an improved binding affinity compared to aptamers obtained via a selection with natural dNTPs (*K*_d_ = 1.1 μM). Moreover, substitution of the modified nucleotides either by wild-type dT units or by a modified dU analog missing the adenine unit led to a marked decrease (~1500 fold less) in binding affinity compared to CMA-59, again highlighting the potential of using base modifications in *in vitro* selection experiments for the generation of aptamers capable of selectively detecting and binding to small molecules. A few years earlier, the same group published a significant paper, where they selected a modified aptamer (using T^HM^TP **8**, Figure 4a) capable of binding only to the (*R*)-isomer of thalidomide (Figure 4b), pointing out the high enantioselectivity modified aptamers can exhibit [115].

**Figure 5:**
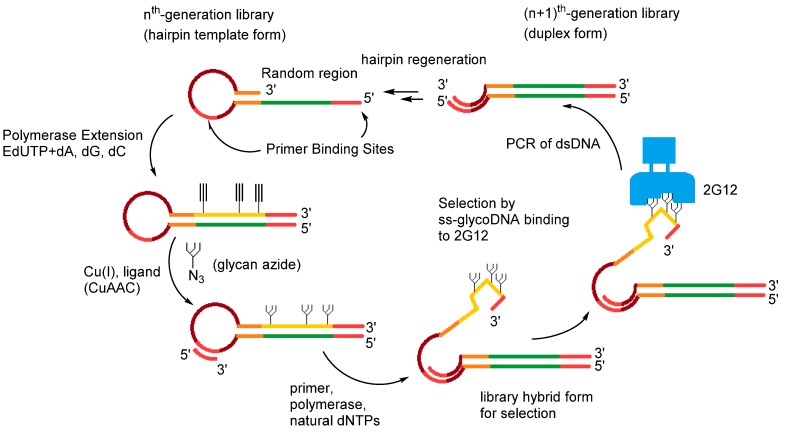
Schematic overview of the SELMA method. Figure adapted from reference [118].

The 5-ethynyl-modified dUTP (EdUTP) was revealed to be a pivotal tool in a variant of *in vitro* selection coined SELMA (SELection with Modified Aptamers) [116]. The alkyne units that are incorporated into the dsDNA hairpin library via primer extension serve as synthetic handles for the grafting of glycans (Man_4_ or Man_9_) onto the nucleic acid scaffold (Figure 5) [117]. The resulting modified glyco-ssDNA strands could then be displaced by a second primer extension reaction using all-natural dNTPs and a primer annealed inside the hairpin loop. This strand displacement also causes a physical separation between phenotype (*i.e.*, the glycol-DNA construct that can act as the aptameric species) and the genotype (*i.e.*, the wild-type dsDNA that encodes the sequence information corresponding to the aptamers) [117]. Subsequently, the modified library is subjected to a standard *in vitro* selection protocol which includes binding to the desired target, PCR amplification of the genotype DNA, and conversion to an enriched population of hairpin ssDNAs (Figure 5). SELMA was employed to identify glycol-DNA aptamers that could recognize the monoclonal antibody 2G12 which is known to bind to mannose-rich glycans on the HIV envelope protein gp120, thus neutralizing various HIV strains [117,118,119]. An initial SELMA experiment using a population with 25% of potential glycan positions in the randomized library and a rather short Man_4_ unit led to the identification of aptamers that bound to 2G12 with dissociation constants in the 200–300 nM range [117]. In order to improve the moderate binding affinities, a SELMA selection that included a longer Man_9_ construct and randomized library that contained fewer potential glycan anchoring sites (7% or 15%) was carried out and resulted in the isolation of a truncated aptamer with an improved binding affinity (*K*_d_ = 150 nM) [118]. Finally, when the selection was carried out at 37 °C rather than at room temperature, but by keeping the same selection set-up, aptamers with binding affinities in the 1.7–16 nM were identified [119]. Interestingly, these aptamers had only three to five glycosylation sites, all of which were necessary for the binding activity, which univocally occurred at the gp120 binding site of 2G12 [119].

Carbohydrates were also the target of a selection experiment that utilized a dUTP analog equipped with a positively charged amino group at the C5 position of the nucleobase (T^HM^TP **8**, Figure 4a), with the intent of increasing the binding affinity to the negatively charged silalyllactose [120]. The modified DNA pool was incubated with biotinylated silalyllactose bound to magnetic particles, and after 13 rounds of SELEX, an aptamer with low binding affinity was isolated (*K*_d_ = 4.9 μM). In a conceptually related experiment, an arginine-modified dUTP was engaged in an *in vitro* selection experiment with the negatively charged glutamic acid as the target [121]. Despite low dissociation constants (in the high micromolar range), the isolated aptamers displayed some enantioselectivity since they could recognize the d- from the l-isomer.

**Figure 6:**
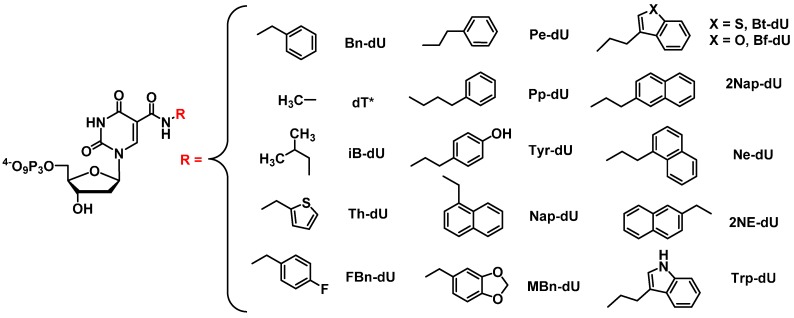
Examples of the 5-modified deoxyuridine triphosphates used as building blocks for the generation of SOMAmers [58,122,125,126]. R groups: Bn-dU: benzyldeoxyuridine; dT*: methyl-dU (thymidine); iB-dU: isobutyldeoxyuridine; Th-dU: 2-thieno-methyl-propyl-deoxyuridine; FBn-dU: 4-fluoro-benzyl-deoxyuridine; Pe-dU: 2-Phenyl-ethyl-deoxyuridine; Pp-dU: 3-Phenyl-*n*-propyl-deoxyuridine; Tyr-dU: tyrosyl-deoxyuridine; Nap-dU: 1-naphtyldeoxyuridine; MBn-dU: 3,4-methyl-dioxy-benzyl-deoxyuridine; Bt-dU: 2-(3-benzo(b)thiophenyl)-ethyldeoxyuridine; 2Nap-dU: 2-naphtyldeoxyuridine; Ne-dU: 2-(1-naphtyl)-ethyldeoxyuridine; 2NE-dU: 2-(2-naphtyl)-ethyldeoxyuridine; Trp-dU: Tryptophanyldeoxyuridine.

Vaught *et al.* synthesized C5-modified carboxamid dUTP derivatives to enable the attachment of a range of hydrophobic groups (for instance, Bn-dU and Trp-dU, see Figure 6) [122]. After testing the enzymatic incorporation of these dU*TPs by polymerases, they selected for modified DNA aptamers against the tumor necrosis factor receptor super family member 9 (TNFRSF9), a protein involved in cancer and inflammatory diseases. The binding affinity of their modified aptamer (*K*_d_ = 0.5–9 nM) surpassed that of a previously published RNA aptamer against the same target (*K*_d_ = 40 nM). It was this work that laid the foundations for the development of novel aptamers termed SOMAmers.

Other various base-modified dN*TPs [123] and N*TPs [124] have been recently evaluated for their capacity at serving in selection experiments, but have not been used for the generation of modified aptamers and thus will not be covered in this review.

#### 2.3.2. SOMAmers

Despite the successful results standard SELEX has presented us with, there are still some protein targets that remain inaccessible to high affinity aptamers as a result of the limited chemical diversity of nucleic acids. What is more, a lower and conservative approach estimates that the human proteome contains at least 10,000 proteins, present at varying concentrations and involved in numerous interactions [127]. In an effort to develop aptamers displaying higher affinities that could be used to explore the human proteome, aptamers were equipped with protein-like side chains with the intent of reducing the k_off_ values (SOMAmers; Slow Off-rate Modified Aptamers) and were shown to tightly and specifically bind to their targets [128]. An impressive array of dN*TPs has been prepared for the generation of SOMAmers and most of these nucleoside triphosphates comprise hydrophobic residues such as naphtyl- or benzyl-groups (Figure 6) which can increase both the number and the strength of hydrophobic interactions between nucleic acids and the corresponding targets, thus partially mimicking the binding mode of antibodies and other proteins. These interactions and the deep involvement of the modified nucleotides were confirmed by crystal structures of several SOMAmer-protein complexes [126,129], and univocally demonstrated by the low- to sub-nanomolar dissociation constants that are regularly observed with SOMAmers [130]. In addition to lowering the *k*_off_ rates (down to ~10^−5^·s^−1^) [130], and thus increasing the affinity and selectivity [131], SOMAmers also present an improved resistance to nucleases [58]. While the selection protocol for SOMAmers largely followed that devised for other modified aptamers, an additional step was included in order to ensure slow *k*_off_ rates. This step involved incubation of the SOMAmer-target protein complexes with a large excess of a non-specific polyanionic competitor (e.g., dextran sulfate) [128].

Finally, those SOMAmers along with novel designed technologies (SOMAscan assay) facilitate the simultaneous detection of various proteins in the blood serum and have been widely applied in the discovery of disease biomarkers [132]. Indeed, within five years, SOMAmers specific for over 3000 human proteins have been identified with unique properties that have the potential to be employed in the fields of therapeutics and diagnostics [58,126].

### 2.4. Spiegelmers

The concept of using l-nucleosides to foster mirror-image aptamers (termed Spiegelmers [133,134]) arose almost 20 years ago, but its impact can be seen at present as three Spiegelmers are currently undergoing clinical investigations. They are synthesized by l-nucleoside phosphoramidites after the corresponding d-aptamer against the mirror-image target of interest has been identified through conventional *in vitro* selection methods [135]. This two-step protocol is necessary since l-nucleoside triphosphates are not accepted by RNA and DNA polymerase [136], a fact that is at the origin of their use as potent antiviral agents [137]. However, progress in polymerase evolution [19,138] and the generation of ribozymes recognizing heterochiral nucleic acids or nucleotides [139], spawn hope for the discovery of enzymes capable of catalyzing the polymerization of L-nucleoside triphosphates which in turn would allow circumventing this protocol. Moreover, due to the enantioselectivity rules that govern nature, Spiegelmers are endowed with high stability against nuclease degradation and make promising drug candidates.

All three Spiegelmers currently in clinical trials have been developed by NOXXON Pharma in Germany [140]. Olaptesed pegol (NOX-A12) is evaluated in two parallel Phase II trials for the treatment of chronic lymphocytic leukemia and refractory multiple myeloma by targeting the CXCL-12 chemokine. Lexaptepid pegol (NOX-H94), an anti-hepcidin l-RNA aptamer, is tested for anemia induced by chronic inflammation, while emapticap pegol (NOX-E36) binds to the monocyte chemoattractant protein 1 (MCP-1) that promotes inflammation in type-2 diabetes.

Apart from the above mentioned Spiegelmers, the group at NOXXON has recently been interested in two additional targets. NOX-G15 is a mixed DNA/RNA mirror-image aptamer that binds to the glucagon and improves the glucose tolerance in models of type 1 and type 2 diabetes [141]. This could ultimately result in decreased insulin demand for the disease. On the other hand, NOX-S93 targets the signaling lipid S1P (sphingosine-1-phosphate), involved in cancer, autoimmune diseases, as well as fibrosis [142]. The l-RNA aptamer showed high affinity (*K*_d_ = 4.3 nM) and good selectivity and successfully inhibited the IGF-1 induced angiogenesis by neutralizing the S1P.

Furthermore, Sczepanski *et al.* developed an l-RNA aptamer to target the HIV-1 trans-activation responsive (TAR) RNA [143]. In this significant work, the authors demonstrated that the strong binding activity (*K*_d_ = 100 nM) and consequently the inhibition of the d-RNA target by this l-RNA aptamer occurred by recognition of a distal loop by means of tertiary interactions rather than Watson-Crick pairing. This mode of interaction could account for even greater specificity thus adding up to the features that make aptamers suitable for therapeutic applications [143]. Consequently, l-RNA aptamers are alluring and promising candidates for therapeutic applications since they confer high binding affinities, good selectivities, high nuclease resistance, and they are not confined to Watson-Crick base-pairing recognition motifs [140].

## 3. XNAs and Expanded Genetic Systems

**Figure 7:**
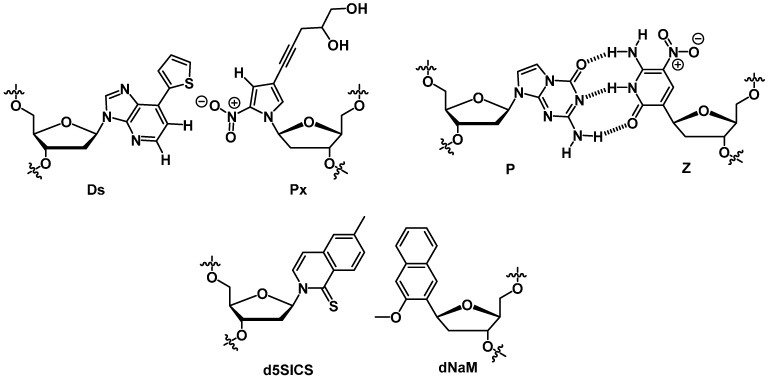
The structures of the unnatural **Ds**:**Px**, **P**:**Z**, and **d5SICS**:**dNaM** base pairs.

The quest for answers regarding the evolution of prebiotic life has brought about a series of studies on the chemical etiology of RNA [144]. In this context, it is believed that the four-letter genetic alphabet evolved as a compromise between replication fidelity, informational complexity, and evolution and adaption capacities [20]. However, expanding the genetic alphabet beyond the canonical Watson-Crick base-pair (and thus increasing the informational complexity) is an alluring goal for the development of functional nucleic acids with improved properties [145,146,147], the creation of systems expressing proteins based on non-standard amino acids [148], and even for the generation of semi-synthetic organisms [149,150]. In a key contribution, Kimoto *et al.* introduced a third base pair in the process of DNA replication by using two unnatural nucleotides (the **Ds**:**Px** pair) that exclusively pair with each other (Figure 7) [146]. The selection experiments yielded aptamers that could bind to VEGF-165 and INF-γ with very high affinity (*K*_d_ values comprised between 1.69 pM and 0.12 nM), one of the few examples where modified aptamers seem to have an improved affinity compared to the natural ones. However, the unnatural base **Ds** had to be placed at predetermined positions in order to facilitate the cloning and sequencing methods, a limitation that could be resolved in the years to come. Similarly, the **Z**:**P** pair (Figure 7), presenting an alternate hydrogen bonding pattern and adopting a Watson-Crick-like geometry [151], was used in an *in vitro* selection experiment using an expanded ACGTZP sequence space for the generation of aptamers that can selectively recognize HepG2 liver cancer cells [152]. Application of 13 rounds of cell-LIVE (laboratory *in vitro* evolution; a variant of SELEX which allows for sequence evolution during the selection process) resulted in the isolation of modified aptamers presenting dissociation constants in the 10–200 nM range. The species bearing **Z**s and **P**s were found to be the better binders compared to unmodified species and the presence of the modified nucleotides was necessary to maintain the high binding affinity [152]. The use of base-modified dN*TPs in the context of an expansion of the genetic alphabet culminated with the creation of an *E. coli* strain where the hydrophobic base-pair system **d5SICS**:**dNaM** (Figure 7) was stably incorporated into a plasmid, and for the first time, resulted in the *in vivo* replication of an unnatural base-pair [150].

**Figure 8:**
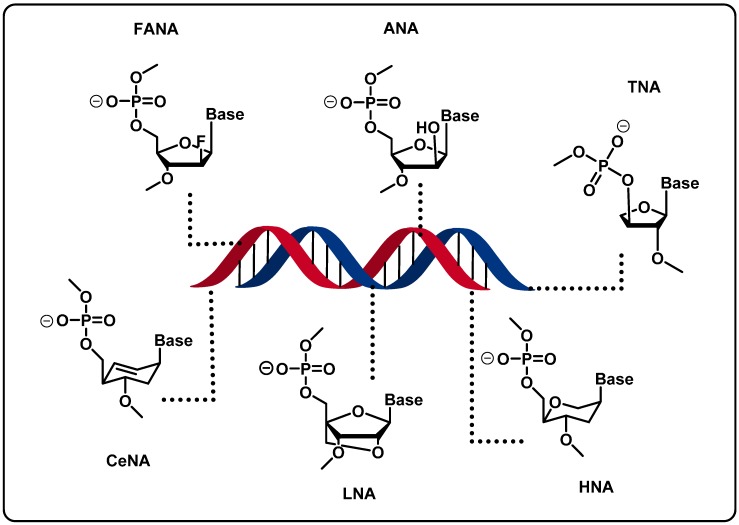
Xeno Nucleic Acids (XNAs) and their structures.

The development of new genetic polymers via Darwinian evolution is not restricted to base-modifications but can also encompass chemical alterations to the sugar moiety [18,19,20]. Indeed, sugar-modifications (*i.e.*, beyond the initial 2′-alterations brought to the ribose unit, *vide supra*) can obviously confer nuclease resistance to oligonucleotides, but, more importantly, can lead to orthogonal artificial nucleic structures (XNAs, Xeno-Nucleic Acids) that are capable of storing information and that support enzymatic synthesis, and thus lead to an expansion of the chemical repertoire (Figure 8) [20]. For instance, the structural key feature of hexitol nucleic acid (HNA) is the 1′,5′-anhydrohexitol backbone, which is at the origin for the versatility of HNA (*i.e.*, stable duplexes are formed with HNA, DNA, and RNA) and the possibility of forming alternate base pairs (e.g., HNA-A/HNA-A) [153]. Recently, an engineered DNA polymerase was shown to support both DNA-dependent HNA polymerization and HNA-dependent DNA polymerization with high fidelity [145]. This fact was then exploited to evolve aptamers against two targets, the trans-activating response RNA (sTAR) and the hen egg lysozyme (HEL) [145]. After eight rounds of *in vitro* selections, HNA aptamers with high affinities for the respective targets were obtained: *K*_d_ values of 28–67 nM when sTAR was the target, and 107–141 nM for anti-HEL aptamers [145]. This approach could also be compatible with other chemistries including FANA, CeNA, ANA, TNA, and LNA modifications of the sugar scaffold (Figure 8) since polymerases accepting these triphosphates as substrates have been engineered (but have not been engaged in aptamer selections) [145]. What is more, the potential of these engineered polymerases to tolerate those modifications was also used to explore catalytic systems, namely XNAzymes catalyzing the scission of ribophosphodiester linkages or acting as ligases [147].

Similarly, TNA nucleoside triphosphates were shown to be good substrates for an engineered polymerase (Therminator DNA polymerase) under primer extension reactions [154,155]. In addition, an *in vitro* selection scheme was devised, where the transcribed TNA population was directly connected to its DNA template, thus connecting phenotype and genotype, respectively [156]. However, some limitations became apparent when a TNA aptamer selection was undertaken: [157] the polymerization reaction comes to a halt when repeating G nucleotides are present in the library and diaminopurine triphosphate should be used instead of TNA-ATP (tATP) to ensure high transcription yields. Notwithstanding these limitations, an anti-thrombin TNA aptamer (*K*_d_ ~ 200 nM) could be selected by using a library that does not contain any cytosine nucleotides, clearly showing that TNAs are capable of Darwinian evolution [157].

As mentioned previously, the LNA structure is another example of sugar modification that has gained significant attention since its advent [158,159]. Due to its nuclease resistance and the unprecedented affinity of LNA oligonucleotides for complementary DNA and RNA sequences, LNA has served as an important tool in many oligonucleotide-based applications including antisense and antigene therapy, diagnostics, and biotechnology [160,161,162]. The compatibility of LNA-triphosphates with RNA- and DNA-polymerases has been demonstrated [163] and was the fundament for the establishment of reliable protocols for *in vitro* selection using this modification [164,165]. Based on these findings, a selection experiment using a single LNA-TTP led to isolation of anti-thrombin aptamers with high binding affinities (*K*_d_ values in the low nM range) [166]. More recently, two LNA aptamers have been proposed, one targeting the VEGF as a potential therapeutic agent against breast cancer [167], whilst the second one aims at the inhibition of cellular CD73, a cell surface protein overexpressed in many solid tumors [168].

Moreover, Hagiwara *et al.* used a random library containing two diverse modifications, namely LNA and 2′-Fluoro nucleic acid (FNA) to select chimeric DNA aptamers by capillary electrophoreses SELEX (CE-SELEX) [169]. In their work, they demonstrated that the DNA strands could adopt an A-type conformation more usually encountered in RNA. Their results confer new paradigms to achieve not only chemical but also structural diversity of nucleic acids.

Finally, nucleoside analogs combining dual base- and sugar-modifications have also been evaluated for their potential to serve in the creation of XNA modified systems and organisms [170]. Indeed, the isoguanine:5-methylisocytosine (isoG:iso^Me^C) pair was grafted on the HNA scaffold and the base-pairing properties and the acceptance of polymerases were determined. These modifications were also studied for their effect on base-recognition and DNA synthesis *in vivo* [170]. Similarly, base-modified LNAs have been shown to be compatible with DNA polymerases under primer extension reaction conditions and with the T7 RNA polymerase to generate RNA transcripts, but have not been used in selection experiments [171].

## 4. Conclusions and Future Directions

Aptamers have emerged as a promising class of therapeutics due to a variety of properties including their small size, the lack of immunogenicity, and their high affinity and selectivity for their targets. Barriers regarding their susceptibility to nucleases have successfully been addressed by the use of modifications post-selection, but in many cases, resulting in a reduced affinity compared to the natural aptamers. An attractive solution to this drawback is the inclusion of modified nucleoside triphosphates directly in the selection experiments. Indeed, this not only circumvents the need for tedious post-selection engineering of the sequences that is often associated with a loss in activity, but also allows for the introduction of functional groups that might increase the affinity by creating new interactions with the target and generate differential folding and structural patterns.

The advent of engineered polymerases that tolerate a broad array of substrates along with recent progress in synthetic organic chemistry has seen a massive expansion of the chemical space that can be explored in SELEX experiments. The positive reflection of these progresses on functional nucleic acids is the emergence of numerous synthetic aptamers with improved properties, including high affinity to the target (*i.e.*, in the low nM to pM range), strong biostability, improved pharmacokinetic properties, recognition of more challenging targets (e.g., glycoproteins and single enantiomers of small organic molecules), and improved bioavailability (e.g., crossing of the BBB or internalization). However, generating modified aptamers with SELEX can sometimes be a more challenging procedure since it is a rather time-consuming process with variable success rates. These technical impediments are partially alleviated by the emergence of new SELEX strategies such as LIVE [172], cell-SELEX [8], or other methodologies for the selection of modified functional nucleic acids [173].

Nevertheless, some aptamers like the SOMAmers have managed to find their way around those issues and reach the industrial threshold encouraging the exploitation of novel structural and functional diversities. So far, the use of modifications in diagnostics has been limited only to the biomarker discovery. It could be interesting to also see their contribution as *in vivo* aptasensors or imaging agents for targets that are inaccessible to natural aptamers.

Taken together, recent progress made in the generation of modified aptamers by selecting with dN*TPs and N*TPs bodes well for the future of aptamers as therapeutic agents and diagnostic tools. The synergy between the design of target-specific modifications on the nucleoside triphosphate(s) and the application of a suitable selection protocol will certainly contribute positively to an increase in the number of aptamers in clinical trials, propelling more modified aptamers to join pegaptanib in the rank of FDA-approved nucleic acid-based drugs.
